# Optimization, Probiotic Characteristics, and Rheological Properties of Exopolysaccharides from *Lactiplantibacillus plantarum MC5*

**DOI:** 10.3390/molecules28062463

**Published:** 2023-03-08

**Authors:** Xuefang Zhao, Qi Liang

**Affiliations:** Functional Dairy Products Engineering Laboratory of Gansu Province, College of Food Science and Engineering, Gansu Agricultural University, Anning District, Lanzhou 730070, China

**Keywords:** *Lp. plantarum MC5*, exopolysaccharides (EPS), antidigestive activity, antioxidant activity, rheology properties

## Abstract

This study optimized the exopolysaccharides (EPS) production for *Lactiplantibacillus plantarum MC5* (*Lp. plantarum MC5*) and evaluated the resistance to human simulated digestive juices, antioxidant activity in vitro, and rheological properties of EPS-*MC5*. The results showed that maximum EPS production of 345.98 mg/L (about 1.5-old greater than the initial production) was obtained at optimal conditions of inoculum size (4.0%), incubation time (30 h), incubation temperature (34.0 °C), and initial pH value (6.40). Furthermore, the resisting-digestion capacity of EPS-*MC5* after 180 min in α-amylase, simulated gastric juice (pH 2.0, 3.0, 4.0), and simulated intestinal juice (pH 6.8) was 98.59%, 98.62%, 98.78%, 98.86%, and 98.74%, respectively. In addition, the radical scavenging rates of DPPH•, ABTS•, •OH, and ferric-iron reducing power (OD_700_) of EPS-MC5 were 73.33%, 87.74%, 46.07%, and 1.20, respectively. Furthermore, rheological results showed that the EPS-*MC5* had a higher apparent viscosity (3.01 Pa) and shear stress (41.78 Pa), and the viscoelastic modulus (84.02 and 161.02 Pa at the shear frequency of 100 Hz). These results provide a new insight into the application of EPS in human health and functional foods, which could also improve theoretical guidance for the industrial application of EPS.

## 1. Introduction

Microorganisms producing bioactive exopolysaccharides (EPS) include archaea, bacteria, and fungi [1]. Lactic acid bacteria (LAB) are well-known EPS-producing bacteria, and their final metabolites are generally recognized as safe (GRAS). They can be applied to different industries, especially the food industry [2] and pharmaceutical industry [3]. As a starter, probiotics are reported to play an important role in the rheology and texture of fermented foods [4]. The widespread use of LAB in baked foods has been reported to improve texture properties, increase flavor and produce lactic acid [5]. EPS also plays an important role in protecting inter-and intra-microbial interactions, serving as a source of nutrients in times of hunger and resistance to virulence when pathogens attack [6]. More than 30 species of LAB have been reported to produce EPS [7], such as *Lactobacillus plantarum* [2], *Lactobacillus rhamnosus* [8], *Streptococcus thermophilus* [9], and so on. Reactive oxygen species (ROS) are used in immune mechanisms to prevent bacterial and viral infections [10]. However, excess ROS can oxidize important biological components such as DNA, lipids, and proteins. This oxidative damage is closely associated with accelerated aging and the development of various diseases, including cancer [11], diabetes [12], and hypertension [13]. Notably, the antioxidant is one of the important prebiotic effects of LAB. The cell surface itself contains antioxidants, and cells also produce antioxidants such as EPS, peptides, and lactic acid [14].

A previous study reported that EPS production and concentration can be affected by medium components (carbon and nitrogen sources) and culture conditions [15]. EPS from LAB can protect cells from desiccation [9], metal ions, antibiotics, bacteriophages, and cell wall-degrading enzymes [16]. Degeest et al. [17] reported that the EPS production and their monomer composition may be affected by the growth medium and culture conditions of laboratory-screened LAB. Likewise, EPS production varies widely (40–1000 mg/L) between strains [7]. Previous literature reported that some EPS-producing *Lp. plantarum* strains isolated from traditional fermented foods possess many functional properties, especially antioxidant activity [18]. *Lp. plantarum* is one of the probiotics that can be used in the food list. Its EPS has good biological activity and industrial application characteristics, but it faces the problem of low yield and instability, which limits its promotion and application. *Lp. plantarum* isolated from traditional fermented dairy products is rare, while *Enterococcus faecalis* is the main strain isolated from traditional fermented dairy products. To improve the commercial-scale production of EPS, response surface methodology was used to model and optimize the fermentation substrate and culture conditions for EPS production [2]. Therefore, it is particularly necessary to strengthen the screening of new strains or optimize EPS production conditions to improve the EPS production of *Lp. plantarum*, which is of great significance for the commercial development of EPS. In addition, although the research on the antioxidant activity of EPS produced by *Lp. plantarum* had some free radical models, they are not systematic and comprehensive.

*Lp. plantarum MC5* was isolated from traditional fermented yak milk in the Gansu Tibetan area, which was identified as *Lactobacillus plantarum* by 16S rRNA sequencing and whole genome sequencing technology. *Lp. plantarum MC5* had better EPS-producing and fermentation capacity [19]. In this study, EPS culture conditions were optimized using response surface methodology (RSM) to increase EPS yield. EPS was prepared and purified under optimum conditions. The probiotic properties of the EPS were further investigated, including anti-α-amylase activity, simulated gastrointestinal fluid tolerance, and antioxidant activities (radical scavenging activity of DPPH•, ABTS•, •OH, and iron-reducing power). In addition, the rheological properties of the EPS were also investigated.

## 2. Results and Discussion

### 2.1. Identification of Strain MC5 and Culture Conditions Single Factor Test

**Figure 1:**
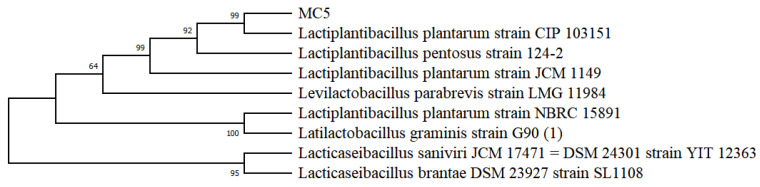
Phylogenetic tree of strain *MC5*.

**Figure 2:**
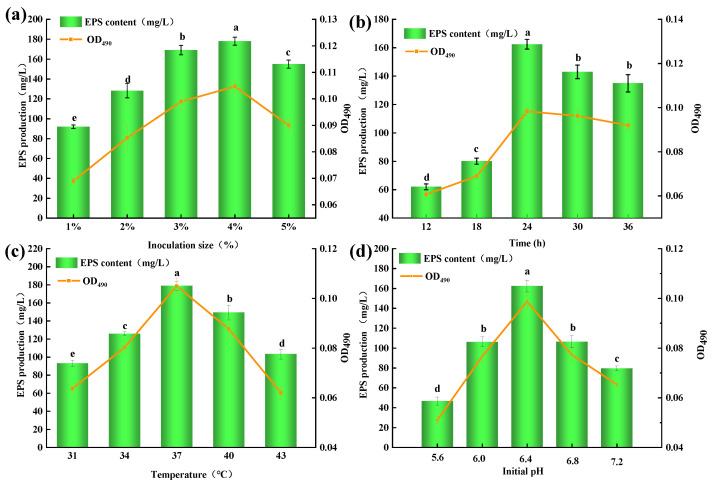
Effect of culture conditions on the EPS production from *Lp. plantarum MC5.* (**a**): Effect of inoculation size on the EPS production from *Lp. plantarum MC5*; (**b**): Effect of culture time on the EPS production from *Lp. plantarum MC5*; (**c**): Effect of culture temperature on the EPS production from *Lp. plantarum MC5*; (**d**): Effect of initial pH on the EPS production from *Lp. plantarum MC5*. Error bars are represented the standard errors (se) of the mean value (n = 3). “a, b, c, d, e” indicate significant differences (*p* < 0.05).

Based on the 16S rRNA sequence, the phylogenetic tree showed the strain *MC5* was more similar to *Lp. plantarum CIP 103151* and *Lp. plantarum JCM 1149* than other species, so the *MC5* was identified as *Lp. plantarum*. (Figure 1). On an industrial scale, the use of microorganisms was beneficial because they can be cultured under controlled conditions and produce large amounts of EPS in a short time [20]. Therefore, culture conditions are important to increase EPS yield and productivity [21]. To improve the EPS production of *Lp. plantarum MC5*, the optimal culture conditions of this strain were studied in this paper (Figure 2).

The results of inoculation size showed that the EPS-*MC5* content increased from 91.98 to 175.45 mg/L at 1–5% (*p* < 0.05, Figure 2a). The maximum EPS content was 175.45 mg/L at 4% inoculation size. Jiang et al. [22] reported that appropriate inoculation size was the prerequisite for high EPS production of microorganisms. The results of fermentation time showed that the EPS content first increased (12–24 h) and then decreased (24–36 h). (Figure 2b). When the fermentation time was 24 h, the maximum EPS yield (164.65 mg/L) was obtained. At this time (24 h), the *Lp. plantarum MC5* reached a stable stage, so the EPS content produced by metabolism was the largest. After 24 h, the EPS content decreased because the large number of metabolites accumulated in the fermentation broth inhibited the growth and reproduction of the strain.

Fermentation temperature results showed that the EPS production increased significantly at the temperature of 31–37 °C (*p* < 0.05), and the EPS content varied in the range of 89.00–186.77 mg/L (Figure 2c). When the temperature was 37 °C, the EPS production reached a maximum of 186.77 mg/L, indicating that the activity of EPS-related enzymes in the cells of *Lp. plantarum MC5* was the highest at this temperature. When the fermentation temperature continued to increase, the EPS production decreased significantly (*p* < 0.05), indicating that the increased temperature inhibited the activities of EPS-related enzymes. Although strain fermentation is a dynamic process, the initial pH of the fermentation broth is also important for EPS production [23]. The EPS production first increased and then decreased at the initial pH of 5.6–7.2 (*p* < 0.05, Figure 2d), indicating that the initial pH was too acidic or too alkaline, which would affect the EPS production of *Lp. plantarum MC5*. When the initial pH was 6.4, the highest EPS production was 169.27 mg/L, indicating that the strain was suitable for growth in a neutral acid environment, and the enzymatic reaction of the strain was more active in this environment.

### 2.2. Optimization of Fermentation Conditions for EPS-MC5

Based on the single factor test, four culture conditions were independent variables, and the EPS production was the response value. Response surface optimization and the test plans were designed by using Design-Expert 8. 0. 6.1 software (Table 1).

The overall quadratic polynomial equation for EPS yield was established by multiple regression analysis on the inoculum size (A), the culture time (B), the culture temperature (C), and the initial pH (D) of *Lp. plantarum MC5*: EPS = 356.92 + 8.38A + 19.15B − 17.79C − 13.39D − 11.25AB + 17.22AC − 0.42AD − 55.91BC + 6.09BD + 4.69CD − 105.37A^2^ − 68.19B^2^ − 79.86C^2^ − 100.95D^2^. The regression model was analyzed by variance analysis and reliability analysis, and the results were shown in Table 2.

The results were subjected to analysis of variance (ANOVA), and statistical tests were performed with the F test shown in Table 2. The F value of the model was 26.34, *p* < 0.0001, indicating that the regression model was statistically significant. The lack of fit term was not significant, indicating that the model had good simulation, and R^2^ and correction coefficient R^2^_Adj_ were 0.9634 and 0.9268, respectively, indicating that the established regression equation had a good degree of fit and could successfully predict the response value. In addition, the coefficient of variation (C.V. = 10.00%) indicated that the experimental results had high precision and reliability. The model data showed that the primary term B, the interaction term BC, A^2^, B^2^, C^2^, and D^2^ had a very significant impact on the EPS yield of the fermentation broth (*p* < 0.01), and the primary term C and D had a significant impact on the EPS yield (*p* < 0.05). To sum up, the order of the significant differences in the influence of the four factors was culture time > culture temperature > initial pH > inoculation size.

### 2.3. Three-Dimensional Response Surfaces and Count Plots of Variables

**Figure 3:**
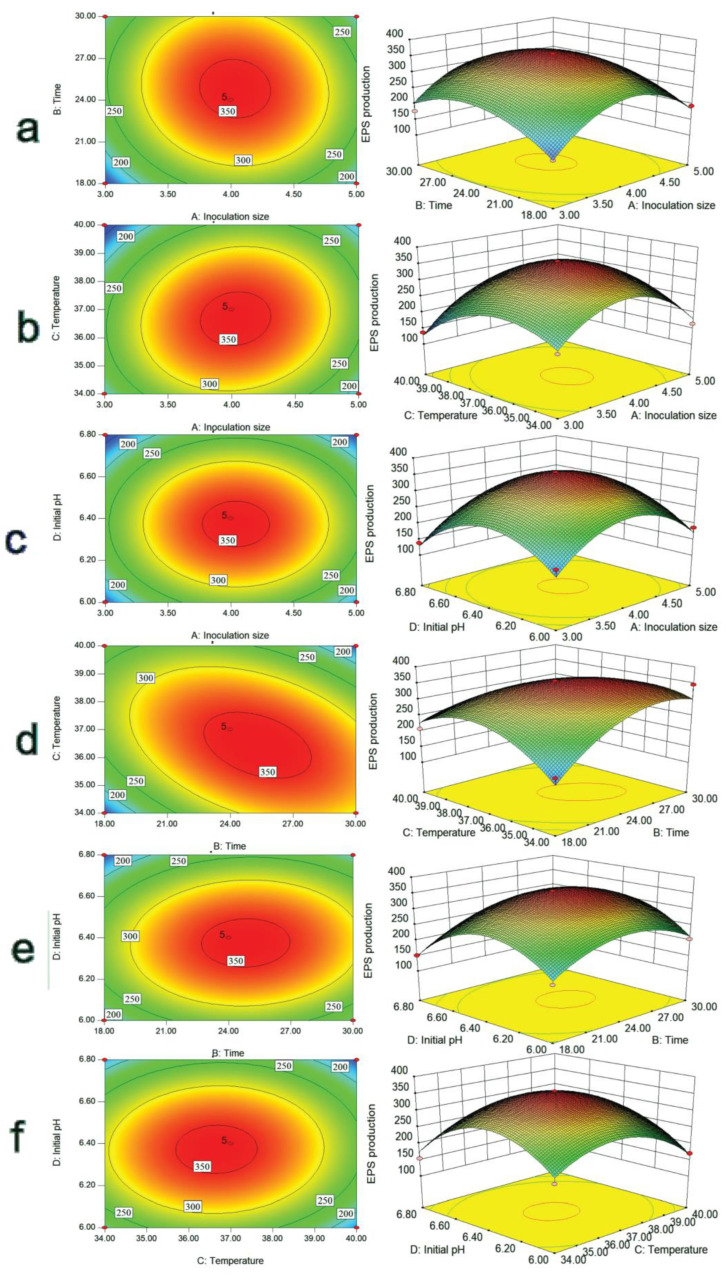
3D response surface plot of the interaction of four factors. (**a**) represents the 3D response surface plot of inoculation size and time; (**b**) represents the 3D response surface plot of inoculation size and temperature; (**c**) represents the 3D response surface plot of inoculation size and initial pH; (**d**) represents the 3D response surface plot of time and temperature; (**e**) represents the 3D response surface plot of time and initial pH; (**f**) represents the 3D response surface plot of temperature and initial pH.

To visualize the effect of the interaction among the four factors of A, B, C, and D on the EPS yield of the strain, the three-dimensional response surface and contour plots between every two factors and EPS yield were drawn (Figure 3). Compared with other graphs, the contour line of the interaction term BC was elliptical, with the densest distribution and the steepest surface, followed by the interaction term AC, which showed that the interaction between culture time, culture temperature, and inoculum size had a more significant effect on EPS yield. This result corresponded to the results in Table 2. Therefore, culture time and temperature were key factors affecting EPS production.

### 2.4. Verification Test of EPS-MC5 Yield

The optimal culture conditions for each factor were predicted as follows: the inoculation size, culture time, culture temperature, and initial pH were 4.24%, 30 h, 34.26 °C, and 6.42, respectively. The predicted EPS yield obtained under these conditions was 355.46 mg/L. In order to verify the effectiveness of the response surface model, considering the possibility of actual operation, the optimal culture conditions were adjusted as follows: the inoculum size was 4.0%, the culture time was 30 h, the culture temperature was 34.0 °C, and the initial pH value was 6.40. Under these culture conditions, the EPS content from *Lp. plantarum MC5* was 345.81 mg/L (Table 3). The experimental results were in good agreement with the predicted results, indicating that the mathematical model was suitable for the simulation of the EPS production process in this study. We verified that the measured EPS yield was close to the EPS yield (356.92 mg/L) obtained by the five groups of parallel experiments at the center point of the response surface.

In addition, the EPS production from other LAB was summarized and compared in Table 4. Many factors affect EPS yield [23,24]. EPS yield and the optimal EPS-producing conditions varied greatly (69–2767 mg/L), which may be due to differences in medium composition, isolation source, and strain species [25].

### 2.5. Isolation and Purification of EPS-MC5

**Figure 4:**
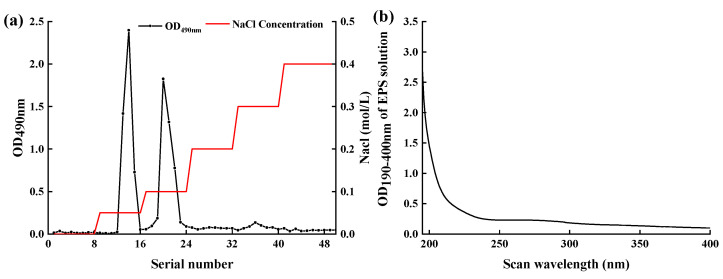
DEAE Sepharose Fast Flow elution curve of EPS-*MC5* (**a**) and UV full wavelength scan of EPS-*MC5* eluent (**b**). Data are represented as the mean values (n = 3).

The EPS-*MC5* was isolated under optimal culture conditions. The crude EPS-*MC5* was separated by anion-exchange chromatography of DEAE Sepharose Fast Flow (Figure 4a). Fractions corresponding to the major peak eluted only with 0.05 Mol/L and 0.1 Mol/L NaCl were found to contain EPS, which showed that 0.05 and 0.1 Mol/L NaCl solution eluted almost all EPS. These fractions containing EPS were acidic EPS as they were eluted with NaCl [29]. The purified EPS eluate was subjected to UV full-wavelength scanning (Figure 4b). EPS-*MC5* had no absorption peaks at 260 and 280 nm, indicating that the nucleic acid and protein in the EPS have been removed, and the EPS had high purity.

### 2.6. In Vitro Resisting-Digestion Capacity of EPS-MC5 to α-Amylase and Simulated Gastrointestinal Juices

The premise of EPS from LAB to play a probiotic role in the human gastrointestinal tract is that it must have a strong anti-digestive ability. Studies have reported that EPS produced by probiotics plays an important role in the colonization of probiotics in the human gastrointestinal tract [30].

**Figure 5:**
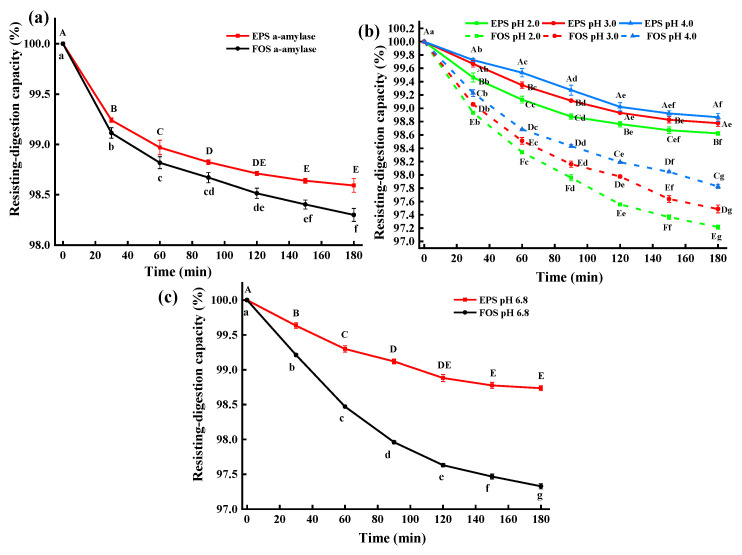
(**a**): The resisting-digestion capacity of EPS-*MC5* in α-amylase solution; (**b**): The resisting-digestion capacity of EPS-*MC5 in* simulative gastric juices (pH 2, 3, and 4); (**c**): The resisting-digestion capacity of EPS-*MC5* in intestinal juices (pH 6.8). Error bars represent the standard errors (se) of the mean value (n = 3). Different lowercase letters indicate the difference between different times of the same pH, different capital letters indicate the difference of different pH at the same time (*p* < 0.05).

At 0–180 min, the α-amylase resisting-digestion capacity (αRC) of EPS-*MC5* was significantly higher than fructooligosaccharides (FOS) (*p* < 0.05). At 0–80 min, the αRC of EPS-*MC5* decreased significantly (*p* < 0.05), while at 80–180 min, the difference in αRC was not significant (*p* > 0.05), indicating that the structure of EPS-*MC5* was stable. The αRC of EPS-*MC5* at 180 min was 98.59% (Figure 5a). The α-amylase can specifically hydrolyze the α-1,4 glucosidic bonds in starch or other polysaccharides. Due to the high αRC of the EPS, the EPS-*MC5* could contain little or no α-1,4 glucosidic bonds.

The resisting-digestion capacity (RC) of EPS-*MC5* was significantly higher than that of FOS in simulated gastrointestinal juices at different pH values (*p* < 0.05), indicating that EPS-*MC5* had a higher anti-digestive ability (Figure 5b,c). Within 0–120 min, the RC of EPS in four different pH simulated gastrointestinal juices decreased significantly (*p* < 0.05), while in 120–180 min, the RC decreased insignificantly (*p* > 0.05). In addition, the RC of the EPS-*MC5* in the simulated gastric juice at pH 2.0 was significantly lower than those in pH 3.0 and pH 4.0 (*p* < 0.05). The results showed that the RC was inversely proportional to pH, with more glycosidic bonds cleavage at low pH. This may be due to that pH affected the RC of EPS by adjusting the enzymatic activity in simulated gastric juice.

The RC of EPS-*MC5* at different pH simulated gastrointestinal juices were 98.62–98.86%. Caggianiello et al. [31] reported that EPS-producing strains are more likely to colonize the intestine because of the adhesion of EPS to intestinal epithelial cells. The results of Mao et al. [32] showed that the EPS produced by *E. coli O157:H7* was resistant to bile salts, and simulated gastrointestinal juices. Devi [30] reported that the hydrolysis resistance of EPS from *Weissella Confusa KR780676* to α-amylase, gastrointestinal juices was 99.1–98.8%, which was close to that of EPS-*MC5* in this study. Results have shown that EPS-*MC5* has good stability to ensure that it works as intended once it enters the intestinal tract.

### 2.7. In Vitro Antioxidant Activity of EPS-MC5

**Figure 6:**
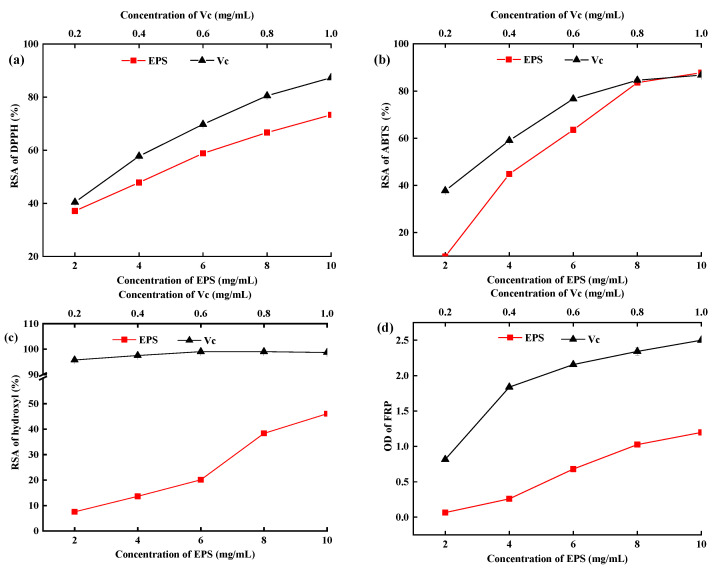
Radical scavenging activity of DPPH (**a**), ABTS (**b**), OH (**c**), and ferric-iron reducing power of EPS-*MC5* (**d**). Ascorbic acid (Vc) was used as a positive control. Error bars represent the standard errors (se) of the mean value (n = 3).

The in vitro radical scavenging rate of EPS is a common method to evaluate the ability and mechanisms of antioxidants [33]. DPPH· is a stable radical with an unpaired electron on one atom of its nitrogen bridge and has a strong absorption band at approximately 517 nm [27,34]. Hydroxyl radical (•OH) has free access to cell membranes and causes tissue damage. Thus, scavenging ·OH may avoid tissue injury [4,35]. The DPPH·, ABTS·, and •OH RSR of the EPS and Vc significantly increased with increasing concentrations (*p* < 0.05, Figure 6a–c). The RSR (DPPH•, ABTS•, and •OH) of EPS increased from 37.12% to 73.33%, 9% to 87.74%, and 7.53% to 46.07% at 2–10 mg/mL concentrations. Miao et al. [36] reported that the RSR of DPPH· was 40% for EPS at 5.0 mg/mL, which supported the results of this study. Results showed that the RSR of DPPH· and ABTS· was higher than that of OH.

The FRP of EPS-*MC5* significantly increased at 2–10 mg/mL (*p* < 0.05, Figure 6d). The highest FRP value of the EPS was 1.24 at 10 mg/mL, which suggested that the EPS not only acted as an electrons donor to directly react with free radicals but also performed antioxidant activity by other mechanisms, for instance, chelating with transition metal ion catalysts [37]. The FRP of EPS from *L. plantarum LP6* was 0.632 [35], which was close to the EPS-*MC5* in this study. The research results of Li [38] also showed that EPS may exert antioxidant activity through multiple mechanisms, such as blocking chain initiation, binding to the transition metal ion catalysts, and decomposing peroxides.

### 2.8. Rheological Properties of EPS-MC5

#### 2.8.1. Apparent Viscosity of EPS-MC5

**Figure 7:**
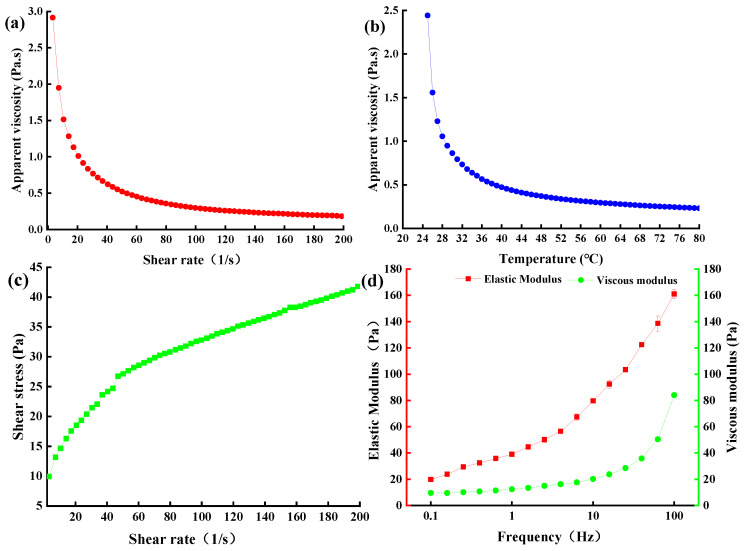
Apparent viscosity of EPS-*MC5* at different shear rates and temperatures (**a**,**b**); shear stress and elastic modulus (G’) and viscous modulus (G”) of EPS-*MC5* (**c**,**d**). Error bars represent the standard errors (se) of the mean value (n = 3).

EPS concentration, processing temperature, pH, and metal ions all affect the rheological properties of EPS [39]. The apparent viscosity decreased gradually with increasing shear rate. When the shear rate increased from 3.24/s to 60/s, the apparent viscosity of the EPS decreased from 3.01 to 0.45 Pa (Figure 7a), which may be due to the disruption of intermolecular interactions and the breaking of bonds between various structural units with the increase of shear rate [39]. EPS from both *L. plantarum C70* [40] and *YW11* [26] also exhibited this shear thinning property. When the shear rate was greater than 60/s, the apparent viscosity decreased slowly and finally stabilized. In addition, the apparent viscosity of EPS-*MC5* decreased rapidly with the increasing shear temperature (Figure 7b). When the shear temperature was 25–60 °C, the apparent viscosity of EPS decreased from 2.44 to 0.29 Pa, while when continuing to increase the temperature, the apparent viscosity of the EPS remained the same.

The shear stress of EPS-*MC5* formed a thixotropic ring, indicating that the EPS was a thixotropic system (Figure 7c). The thixotropic ring of the EPS was large, indicating that the EPS had large stress under the action of external force. When the shear rate was 3.24–200/s, the stress of EPS-*MC5* increased from 9.93 to 41.78 Pa.

#### 2.8.2. Viscoelastic Properties of EPS-MC5

Viscoelasticity is one of the important processing characteristics of EPS [40]. The elastic modulus (G’) was generally higher than the viscous modulus (G”, Figure 7d), indicating that the EPS itself formed a gel structure and had elastic and solid-like characteristics. With the increasing frequency (0.1–100 Hz), the viscoelasticity modulus of EPS-*MC5* increased from 19.81 to 161.02 and 9.63 to 84.02 Pa, respectively. This showed that the work done by the outside world on EPS aggregates increased at high frequencies. However, the viscoelasticity of EPS-M41 at 0.1–10 Hz was 1.0 × 10^−5^–1.0 Pa [40], which was lower than that of EPS-*MC5* in this study. These differences may be due to the different molecular weights, glycosidic bond type, monosaccharide composition, functional groups, and substituents [41].

## 3. Materials and Methods

### 3.1. Materials

The strain *Lp. plantarum MC5* was isolated from traditional fermented yak milk samples, in Tibetan areas of Gansu, China. Strain *MC5* was identified by 16S rRNA sequencing and whole genome sequencing. *Lp. plantarum MC5* was maintained in MRS agar dishes at 4 °C for immediate use and prepared to skim milk and glycerol stocks for a long time of preservation in a deep freezer (−80 °C). α-amylase (8 U/mg) was supplied by Shanghai Macklin Biochemical Co., Ltd. (Shanghai, China). Pepsin and Trypsin were supplied by Beijing Solebro Science and Technology Co., Ltd. (Beijing, China). All reagents used were of analytical grade.

MRS broth [42]: peptone (10 g/L), beef extracts (10 g/L), yeast extract (5 g/L), glucose (25 g/L), Tween 80 (1 mL/L), K_2_HPO_4_ (2 g/L), sodium acetate (5 g/L), diammonium hydrogen citrate (2 g/L), MgSO_4_ (0.2 g/L), MnSO_4_ (0.08 g/L), and agar (15 g/L). It was sterilized at 121 °C for 20 min.

### 3.2. Isolation and Determination of EPS-MC5

*Lp. plantarum MC5* was cultured in MRS broth for 24 h, and the supernatant was collected after centrifugation. The supernatant was mixed with 80% (*w*/*v*) trichloroacetic acid (TCA), allowed to stand at 4 °C for 24 h, and then centrifuged again. The supernatant was collected, mixed with 95% alcohol (3:1 *v*/*v*), and allowed to stand again at 4 °C for 24 h. The mixture was centrifuged, and the pellet was suspended in deionized water and dialyzed at 4 °C for 2 days using dialysis bags (molecular weights of 8–14 kDa). The total sugar content was detected using phenol-sulfate acid method [28]. Briefly, 0.1 g EPS was dissolved in 100 mL of distilled water, and 1 mL EPS solution was mixed well with 1 mL 6% (*w*/*v*) phenol solution and 5 mL 98% (*w*/*v*) H_2_SO_4_, shaken for 10 min, and the absorbance was determined at 490 nm.

3,5-dinitro salicylic acid (DNS) method was utilized to detect the reducing sugar content [43]. A total of 2 mL distilled water and 1.5 mL DNS solution were added to 0.2, 0.4, 0.6, 0.8, 1.0, 1.2, and 1.4 mL of 1.0 mg/mL glucose standard solution, respectively. They were shaken well and heated in a 100 °C water bath for 5 min. Then we adding distilled water to make up to 25 mL, and the absorbance was determined at 520 nm.

EPS content (mg/L)=The total sugar content −The reducing sugar content

### 3.3. Purification of EPS-MC5

The EPS was purified by using the method of Zhang et al. [25]. The crude EPS solution (20 mg/mL, 5 mL) was fractionated with an anion exchange chromatography on a DEAE Sepharose Fast Flow column (16 mm × 25 cm), eluted with deionized water, 0.1, 0.2, 0.3, 0.4, and 0.5 Mol/L NaCl solution at a flow rate of 1 mL/min. Every 7 mL elution was collected automatically and the EPS content was determined by the phenol-sulfuric acid method. Peak fractions containing EPS were pooled, dialyzed, and lyophilized.

### 3.4. Single Factor Experiment of Culture Conditions for EPS Production from Lp. plantarum MC5

#### 3.4.1. Effects of Inoculation Size on EPS Production from *Lp. plantarum MC5*

*Lp. plantarum MC5* was inoculated in MRS broth (pH 6.4). The inoculation size of strains *Lp. plantarum MC5* was 1%, 2%, 3%, 4%, and 5%, respectively. They were incubated at 37 °C for 24 h. EPS production was determined by the method in Section 2.2.

#### 3.4.2. Effects of Culture Time on EPS Production from *Lp. plantarum MC5*

*Lp. plantarum MC5* was inoculated into MRS broth (pH 6.4). The *Lp. plantarum MC5* were incubated at 37 °C for 12, 18, 24, 30, and 36 h, respectively. The method of determining EPS production was the same as above.

#### 3.4.3. Effects of Culture Temperature on EPS Production from *Lp. plantarum MC5*

*Lp. plantarum MC5* was inoculated into MRS broth (pH 6.4). The *Lp. plantarum MC5* were incubated at 31 °C, 34 °C, 37 °C, 40 °C, and 43 °C for 24 h, respectively. The method of determining EPS production was the same as above.

#### 3.4.4. Effect of Initial pH Value on EPS Production from *Lp. plantarum MC5*

*Lp. plantarum MC5* was inoculated into MRS broth. The initial pH values of the medium were adjusted to 5.6, 6.0, 6.4, 6.8, and 7.2, respectively, which were incubated at 37 °C for 24 h (inoculation size 4%). The method of determining EPS production was the same as above.

### 3.5. Optimization of Lp. plantarum MC5 EPS Culture Conditions by Response Surface

Using Design Expert 8.0.6.1 Box–Behnken Design (BBD) software to design experiments and analyze the experimental data. The second-order response surface model was obtained by fitting, and the optimal conditions were determined. BBD was experimented to optimize the four variables (inoculation size, incubation time, incubation temperature, and initial pH) screened by single factor experiments and designed as A, B, C, and D. The four independent variables were investigated at three levels with 29 experimental runs and 5 repetitive central points.

The experiments were carried out in triplicates. The 3D graphic plots obtained by the software would illustrate reciprocal interactions between each significant factor [4].

### 3.6. In Vitro Resisting-Digestion Capacity of EPS-MC5

#### 3.6.1. The Resisting-Digestion Capacity of EPS-MC5 to α-Amylase (RCA)

The RDCA of EPS-*MC5* was performed as described by Al-Sheraji et al. [44]. A total of 500 mg EPS was dissolved with 50 mL PBS buffer (pH 6.8), and a-amylase was added to the EPS solution to a final concentration of 8 U/mL. The solution was reacted in a water bath at 37 °C for 180 min. The released reducing sugar content of EPS solution was determined every 30 min, which was detected by the DNS method. The initial total sugar was detected by the phenol-sulfuric acid method. The resisting-digestion capacity (RC) was calculated according to the formula:

$$\mathrm{RC}(\%)=(1-\frac{\mathrm{Hydrolyzed}\;\mathrm{reducing}\;\mathrm{sugar}\;\mathrm{content}}{\mathrm{Total}\;\mathrm{sugar}\;\mathrm{content}-\mathrm{Initial}\;\mathrm{reducing}\;\mathrm{sugar}\;\mathrm{content}})\times 100\%$$

#### 3.6.2. The Resisting-Digestion Capacity of EPS-MC5 to Simulated Gastric Juice

Artificial simulated gastric juice [45]: 3.0 g pepsin was fully dissolved in 300 mL normal saline, and divided into 3 equal parts. Then, the three solutions were adjusted the pH to 2.0, 3.0, and 4.0 with HCl (2 Mol/L), respectively. Finally, the three solutions were filtered and sterilized by using a microporous membrane (0.22 μm).

A total of 500 mg EPS-*MC5* was added to 50 mL of simulated gastric juice with pH 2, 3, and 4, respectively. The three solutions were reacted in a water bath at 37 °C for 180 min. The released reducing sugar content of the EPS solution was determined every 30 min [30]. The calculation formula of the RC was the same as in Section 3.6.1.

#### 3.6.3. The Resisting-Digestion Capacity of EPS-MC5 to Simulated Intestinal Juice

Artificial simulated intestinal juice [46]: Ox bile salt (3.0 g), ox bile juice (1.0 g), and trypsin (2.0 g) were added in PBS buffer (500 mL pH 6.0). Then, the solution was adjusted the pH to 6.8 with NaOH (2 Mol/L). Finally, the solutions were filtered with a microporous membrane (0.22 μm) for later use.

A total of 500 mg EPS-*MC5* was added to 50 mL of simulated intestinal juice with pH 6.8. The solutions were reacted in a water bath at 37 °C for 180 min. The released reducing sugar content of the EPS solution was determined every 30 min [30]. The calculation formula of the RC was the same as in Section 3.6.1.

### 3.7. In Vitro Antioxidant Activity Analysis of EPS-MC5

#### 3.7.1. The Radical Scavenging Rate (RSR) of DPPH

RSR of DPPH free radical was assayed by El-Dein’ method [28]. A total of 2 mL of EPS solution (2, 4, 6, 8, and 10 mg/mL) was mixed with 2 mL 0.1 mMol/L DPPH solution. Then, the mixture was placed in the dark at room temperature for 30 min. The absorbance of the supernatant was determined at 517 nm after centrifugation (A_j_), and ascorbic acid was used as a positive control. The RSR of DPPH free radical was calculated by the equation as follows:

$$\mathrm{Scavenging}\;\mathrm{activity}(\%)=(1-\frac{A_{j}-A_{i}}{A_{0}})\times 100\%$$

A_j_: Absorbance of EPS solution (2 mL) + 95%-DPPH ethanol solution (2 mL);

A_i_: Absorbance of EPS solution (2 mL) + 95% ethanol solution (2 mL);

A_0_: Absorbance of 95%-DPPH ethanol solution (2 mL) + 95% ethanol solution (2 mL).

#### 3.7.2. The Radical Scavenging Rate (RSR) of ABTS

ABTS solution was prepared by mixing equal volumes of ABTS (7 mMol/L) and potassium persulfate solutions (2.45 mMol/L), and the mixture was placed in the dark for 16 h [4]. The ABTS solution was diluted by PBS solution (0.2 M, pH 7.4) to an absorbance of 0.70 ± 0.02 at 734 nm before use. A total of 600 uL of EPS solution (2, 4, 6, 8, and 10 mg/mL) was added into 3 mL ABTS solution. Then, the mixture was incubated for 10 min in dark at room temperature. The absorbance of the mixture solution was determined at 734 nm (A_j_), and ascorbic acid was used as a positive control. The RSA of ABTS was calculated using:

$$\mathrm{Scavenging}\;\mathrm{activity}(\%)=(1-\frac{A_{j}-A_{i}}{A_{0}})\times 100\%$$

A_j_: Absorbance of EPS solution (600 uL) + ABTS solution (3 mL);

A_i_: Absorbance of EPS solution (600 uL) + deionized water (3 mL);

A_0_: Absorbance of deionized water (600 uL) + ABTS solution (3 mL).

#### 3.7.3. The Radical Scavenging Rate (RSR) of Hydroxyl

The RSR of Hydroxyl (OH) was investigated by the method of Zhang [25] with slight modifications. One mL of EPS solution (2, 4, 6, 8 and 10 mg/mL) was mixed with 1.8 mM FeSO_4_ (2 mL), 1.8 mM salicylic acid (1.5 mL) and 0.3% H_2_O_2_ (2 mL). After 30 min of standing at 37 °C and centrifugation (8000 rpm, 5 min), the absorbance of the supernatant was measured at 510 nm. Ascorbic acid was used as a positive control. The RSA of OH was calculated using:

$$\mathrm{Scavenging}\;\mathrm{activity}(\%)=(1-\frac{A_{j}-A_{i}}{A_{0}})\times 100\%$$

A_j_: Absorbance of EPS solution + H_2_O_2_;

A_i_: Absorbance of deionized water + H_2_O_2_;

A_0_: Absorbance of salicylic acid was replaced by deionized water.

#### 3.7.4. The Ferric-Iron Reducing Power (IRP) of EPS-MC5

The IRP of EPS was determined according to the method of Wang [47] with some modifications. Briefly, 1 mL of EPS solution (2, 4, 6, 8 and 10 mg/mL) was mixed with 2.5 mL phosphate buffer (0.2 Mol/L, pH 6.6) and 2.5 mL potassium ferricyanide (1%, *w*/*v*), and the mixture was incubated at 50 °C for 20 min. After adding 2.5 mL trichloroacetic acid (10%, *w*/*v*), the mixture was centrifuged at 8000 rpm for 5 min. A total of 2.5 mL of supernatant was collected and mixed with 2.5 mL distilled water and 0.5 mL FeCl_3_ (0.1%, *w*/*v*). The absorbance was measured at 700 nm after 10 min. The IRP of EPS was calculated using:

OD =Absorbance of sample

### 3.8. Analysis of Rheological Properties of EPS-MC5

#### 3.8.1. The Preparation of the EPS-MC5 Samples

*Lp. plantarum MC5* was cultured in MRS broth for 30 h, and the supernatant was collected after centrifugation. The supernatant was mixed with 80% (*w*/*v*) trichloroacetic acid (TCA), and allowed to stand at 4 °C for 24 h. The supernatant was collected after centrifugation again, mixed with 95% alcohol (3:1 *v*/*v*), and allowed to stand again at 4 °C for 24 h. The EPS-*MC5* samples were obtained after centrifugation.

#### 3.8.2. Apparent Viscosity and Flow Curves of EPS-MC5

The apparent viscosity and shear stress were determined according to Ayyash’s method [40]. Apparent viscosity and shear stress of the EPS-*MC5* samples were determined using an MCR301 Rheometer. The samples were linearly sheared at a constant temperature of 25 ± 1 °C. They were performed in the shear rate range of 0.1 to 200/s. The measurement time was 1 min. In addition, the apparent viscosity of EPS-*MC5* was analyzed as a function of temperature from 25 °C to 80 °C. The temperature ramp rate was 1 °C/min at a constant shear rate of 20/s.

#### 3.8.3. Amplitude and Frequency Sweep Tests of EPS-MC5

The viscoelasticity of the EPS-*MC5* samples was measured by using an MCR301 Rheometer [48]. The frequency sweep test was used to evaluate viscoelasticity of the EPS-*MC5* under the condition of 5% strain force and frequency of 0.1–100 Hz.

### 3.9. Statistical Analysis

All the experiments were carried out in triplicate. The response surface analysis was obtained by using Design-Expert 8.0.6.1 software. The relative standard error and the mean values were calculated using SPSS 22.0 (Statistical Package for the Social Sciences, Chicago, IL, USA). ANOVA tests were done to a determination of significant differences between treatments with a level of significance of *p* < 0.05 by the SPSS 22.0 package program. The obtained pictures were carried out according to the Origin 8.0 software (Statistical Package for the Social Sciences, Northampton, MA, USA).

## 4. Conclusions

In this study, the optimal EPS-producing conditions (the inoculum size of 4.0%, the culture time of 30 h, the culture temperature of 34.0 °C, and the initial pH value of 6.40) and the maximum EPS yield (345.81 mg/L) of *Lp. plantarum MC5* were obtained. The resistance of EPS-*MC5* to human simulated digestive juices (α-amylase, simulated gastric and intestinal juices) was significantly higher than FOS (*p* < 0.05), indicating that EPS-*MC5* could reach the gastrointestinal tract smoothly when entering the human body. The EPS-*MC5* also had high antioxidant activity and could scavenge a variety of radicals (DPPH•, ABTS•, and •OH), which indicated that it exerted antioxidant activity through multiple pathways. In addition, EPS demonstrated good apparent viscosity and viscoelasticity, indicating that it had good processing characteristics.

In conclusion, the EPS-*MC5* was a potential active polysaccharide that could regulate human health and can be used in the food processing industry. However, the structural characterization and beneficial nature in vivo of the EPS-*MC5* need further study.

## Figures and Tables

**Table 1:** Design and results of response surface experiments. Data are represented as the mean (n = 3).

Run	Factor 1 (%) Inoculation Size	Factor 2 (h) Time	Factor 3 (°C) Temperature	Factor 4 Initial pH	EPS (mg/L)
1	4	24	37	6.40	356.92
2	5	24	37	6.00	187.33
3	4	24	34	6.80	154.49
4	4	24	37	6.40	356.92
5	5	18	37	6.40	194.38
6	4	24	37	6.40	356.92
7	5	24	34	6.40	165.39
8	4	24	34	6.00	194.61
9	3	24	40	6.40	136.76
10	3	30	37	6.40	177.21
11	3	24	34	6.40	188.13
12	4	24	37	6.40	356.92
13	4	18	34	6.40	171.54
14	4	30	40	6.40	158.81
15	3	24	37	6.80	139.14
16	4	30	37	6.80	205.19
17	4	18	37	6.80	151.87
18	5	24	37	6.80	148.58
19	4	24	37	6.40	356.92
20	4	24	40	6.00	210.74
21	4	24	40	6.80	169.37
22	3	24	37	6.00	176.22
23	5	30	37	6.40	183.02
24	4	18	40	6.40	208.02
25	4	30	37	6.00	224.72
26	3	18	37	6.40	143.59
27	4	18	37	6.00	175.74
28	4	30	34	6.40	345.98
29	5	24	40	6.40	182.90

**Table 2:** Variance analysis of regression model.

Source	Sum of Squares	df	Mean Squares	F-Value	*p*-Value	Significance
Model	1.629 × 10^5^	14	11,638.92	26.34	<0.0001	Significant
A-Inoculation size	842.53	1	842.53	1.91	0.1890	
B-Time	4400.29	1	4400.29	9.96	0.0070	**
C-Temperature	3799.94	1	3799.94	8.64	0.0109	*
D-Initial pH	2152.58	1	2152.58	4.87	0.0445	*
AB	505.80	1	505.80	1.14	0.3028	
AC	1186.11	1	1186.11	2.68	0.1236	
AD	0.70	1	0.70	1.578 × 10^-3^	0.9689	
BC	12,504.83	1	12,504.83	28.30	0.0001	**
BD	148.11	1	148.11	0.34	0.5718	
CD	87.89	1	87.89	0.20	0.6624	
A^2^	72,014.98	1	72,014.98	162.97	<0.0001	**
B^2^	30,161.36	1	30,161.36	68.26	<0.0001	**
C^2^	41,364.46	1	41,364.46	93.61	<0.0001	**
D^2^	66,101.51	1	66,101.51	149.59	<0.0001	**
Residual	6186.39	14	441.88			
Lack of fit	6186.39	10	618.64			
Pure error	0.000	4	0.000			
Cor total	1.691 × 10^5^	28				
R^2^	0.9634					
Adj-R^2^	0.9268					
C.V. %	10.00					

Note: * means significant difference (*p* < 0.05), ** means extremely significant difference (*p* < 0.01).

**Table 3:** EPS production before and after *Lp. plantarum MC5* optimized culture conditions.

Groups	OD_600_	EPS (mg/L)
Verification value	0.077 ^a^	345.98 ^A^
Initial value	0.065 ^b^	140.34 ^B^

Data are represented as the mean ± standard errors (n = 3). Different letters indicate significant differences (*p* < 0.05).

**Table 4:** EPS production of other lactic acid bacteria.

Strains	EPS (mg/L)	DPPH	OH	Isolation Source
*L. plantarum C88*	69.00	52.23% (4000 mg/L)	85.21% (4000 mg/L)	Chinese traditional fermented dairy Tofu [25]
*L. plantarum YW11*	90.00	-	-	Kefir grains collected from Tibet [26]
*L. plantarum EP56*	126.40	-	-	Corn silage [15]
*S. thermophilus W22*	127.00	-	-	Village type yogurt [9]
*L. delbruckii* subsp. *Bulgaricus B3*	263.00	-	-	
*L. delbruckii* subsp. *Bulgaricus G12*	238.00	-	-	
*L. rhamnosus ATCC 9595*	352.00	-	-	Human breast milk [27]
*L. rhamnosus SHA114*	461.00	-	-	
*L. rhamnosus SHA113*	549.60	-	-	
*L. plantarum KX041*	599.52	82.00% (6000 mg/L)	82.64% (8000 mg/L)	Traditional Chinese pickle juice [24]
*L. plantarum NTMI20*	827.00	91.86% (500 mg/L)	-	Milk sources [2]
*L. plantarum NTMI05*	956.00	96.62% (500 mg/L)	-	
*L. plantarum KU985433*	2030.00	88.00% (4000 mg/L)	-	Egyptian fermented food [28]
*L. rhamnosus RW-9595 M*	2767.00	-	-	LAB research network culture collection [8]

## Data Availability

The data presented in this study are available upon request from the corresponding author. The data are not publicly available due to privacy.

## Abbreviations

EPS	Exopolysaccharide
EPS-*MC5*	Exopolysaccharide production from *Lp. plantarum MC5*
LAB	Lactic acid bacteria
*Lp. plantarum MC5*	*Lactiplantibacillus plantarum MC5*
RSA	Radical scavenging activity
DPPH•	1,1-Diphenyl-2-picrylhydrazyl, (free radical)
ABTS•	2,2’-Azinobis (3-ethylbenzothiazoline-6-sulfonic acid ammonium salt)
•OH	Hydroxyl (free radicals)
IRP	Ferric-iron reducing power
